# Editorial: Exploring bereavement and public health: the role of family and friend caregivers in community well-being

**DOI:** 10.3389/fpubh.2026.1795849

**Published:** 2026-02-18

**Authors:** Toni P. Miles, Wendy G. Lichtenthal, Clare Killikelly, Lauren J. Breen, Don Lollar

**Affiliations:** 1 Carter Center, Mental Health and Caregiver Program, Atlanta, GA, United States; 2 University of Georgia, Athens, GA, United States; 3 University of Miami Miller School of Medicine, Miami, FL, United States; 4 Universitat Zurich, Zurich, Switzerland; 5 Curtin School of Population Health and Curtin enAble Institute, Curtin University, Perth, WA, Australia; 6 Professor Emeritus, Department of Public Health, Oregon Health & Sciences University, Portland, OR, United States

**Keywords:** bereavement–life change events–prevention, caregivers, culture and mental health, policy, population, prolonged grief disorder (PGD), public health

The circle of bereavement after the deaths of family and friends broadly influences societal health (1, 2). As yet, no public health surveillance systems quantify bereavement at population or geographic scales. This is a huge gap in our understanding of population health factors. Consider the course of bereavement among family caregivers. They are a group who is largely unseen in their grief, both before and after the death of the care recipient. With an intentional research focus on bereavement, their needs as well as those of the broader society could be met with effective and timely care for the emotional distress and declines in physical health. Bereavement is a global issue shaped by cultural and local practices (3). The support of bereaved populations requires a broader frame for research questions, study designs that incorporate qualitative and quantitative methods, and a longitudinal perspective. This collection of papers is designed to encourage scaling by showing research designed to fill these gaps. With this Research Topic “Exploring bereavement and public health: the role of family and friend caregivers in community well-being”, we recruited studies that highlight the public health implications of bereavement beyond individual grief. As seen through the lens of caregiver bereavement, public health must develop strategies to overcome the challenges faced by caregivers and their communities and address their underrepresentation in the work of researchers, funding agencies, and policymakers (4). To achieve this goal, we sought to draw attention to the essential roles of caregivers and emphasize the impacts of caregiving and bereavement on caregivers' mental, physical, and social well-being. In doing so, we hope to amplify the importance of efforts to enhance the overall well-being of caregivers and create stronger support networks for those experiencing the loss of the persons in their care.

In recognition of this reach, Frontiers in Aging & Public Health partnered with Frontiers in Public Mental Health to promote this series. Grief is universal, and thus an international matter. This recognition formed the basis for selection of the articles selected for inclusion. The eight articles in this collection provide readers with a glimpse of this broader landscape. A second volume is currently under development. Forty-five authors from across the globe contributed their research. The larger community showed their interest in this topic with 29,876 article views and 3,794 downloads to date.

Weathers et al. showed that 54% of 2,259 adults in Ireland surveyed in 2021 and 2022 reported having experienced one or more losses during the COVID-19 pandemic. Of these, 14% met criteria for prolonged grief disorder and 26% indicated sub-threshold prolonged grief disorder, leading the authors to conclude that the findings provide support for public health models of bereavement care.

Nielsen and colleagues measured prolonged grief symptoms in a sample of 1,735 adults in Denmark who were bereaved by the death of a relative over a period of 10 years. Compared to a low grief trajectory group (45%), a high grief trajectory group (6%) showed persistent high grief symptom levels and had significantly more general practitioner appointments, mental health service use, prescription medication use, and excess mortality.

Tognela et al. interviewed 16 bereaved parents in Australia about their social support experiences—including when it goes well and when it doesn't—showing that social support is a dynamic, relational, and subjective process where attunement between the supporter and recipient matters. The findings align with concern about approaches that uncritically positioned bereavement care as a community responsibility without acknowledgment that community members are not always equipped to provide such support (1).

Schwind et al. provide a multidisciplinary, multi-national perspective of family caregiving and wellbeing in adult chronic illness. In drawing on their scholarly expertise in Australia, Germany, and Switzerland, the authors outline a compelling argument for reimaging research and practice so that that the wellbeing of families, structural enables and barriers, and broader family and social contexts, become central areas of focus.

In a systematic review, Cui et al. conducted a qualitative meta-synthesis of studies to explore the experiences and needs of family members following perinatal infant deaths. The researchers identified 10 studies from nine countries that together showed several unmet needs requiring comprehensive, tailored support strategies.

Andriessen et al. interviewed 34 men bereaved by suicide in Australia. The findings showed the profound, multifaceted impacts of suicide bereavement, including disruptions to close relationships and caregiving roles such as being a full and present parent.

Tay et al. conducted a retrospective cohort study of linked health facility records of 1,224 dyads comprising deceased lung cancer patients and their bereaved spouses in the United States over an 8-year period. After controlling relevant covariates, analysis showed that spouses with preexisting mental health conditions were over four times more likely to develop mental health conditions following bereavement compared to spouses with no preexisting mental health diagnoses.

Finally, Chen et al. used data from the China Health and Retirement Longitudinal Study to investigate the trajectories of depressive symptoms associated with child bereavement among older adults in China. Analysis showed the presence of four trajectories of depressive symptoms associated with child bereavement: low depression that rapidly increases (12%), high depression that rapidly declines (12%), high depression that slowly increases (23%), and stable low depression (53%).

The International Alliance of Carer Organizations—representing 17 countries—reported an estimated 251.5 million persons are engaged in family caregiving (5). In addition to family caregivers, individuals employed in public safety, public health, and other professional fields are also exposed to high rates of deaths and dying. These are individuals who are particularly vulnerable to the health risks associated with bereavement. Building on this first volume, a second volume is planned, which will continue the focus on caregiving while broadening the umbrella of populations to include professional caregivers and increasing the scope of associated domains of investigation. To guide clinical care and policy support, it is essential to expand the research focus on bereavement.
